# Pushing your luck: on chance, serendipity, and athlete development

**DOI:** 10.1186/s40798-025-00932-8

**Published:** 2025-11-21

**Authors:** Joseph Baker, Kathryn Johnston

**Affiliations:** Tanenbaum Institute for Science in Sport, Faculty of Kinesiology and Physical Education, University of Toronto, Toronto, Canada

**Keywords:** Randomness, Athlete performance, Sport, Athlete forecasting, Athlete development, Talent

## Abstract

The notion that problems with prediction can be resolved with more, and better, data has a long history. In this paper, we examine the role of chance and randomness (i.e., events where there is a low probability of occurrence) in athlete development, focusing on the influence of ‘luck’ on this process. More specifically, we briefly summarize the way luck has been considered in previous research on human achievement and how different types of luck (i.e., luck related to elements of the task, the athlete development environment, and biological processes) can affect athlete development. In addition, the implications and challenges of embracing the influence of luck on models of athlete development are discussed. Acknowledging the role of luck may lead to developmental environments that are more equitable (e.g., by creating greater opportunities for more individuals to get lucky) *and* realistic (i.e., by acknowledging that predictions of sport- and athlete-related outcomes will never be perfect).

## Key Points

1. Discussions of athlete development rarely consider the role of random influences.
2. Integrating elements of ‘luck’ into models of athlete development may promote approaches that are less rigid and constrained.
3. Less rigid models of athlete development may lead to more athletes engaging for longer in competitive systems, and improvements to athlete development outcomes.

## Introduction

*In a universe of electrons and selfish genes, blind physical forces and genetic replication, some people are going to get hurt, other people are going to get lucky, and you won't find any rhyme or reason in it, nor any justice.* Richard Dawkins [1].

Scientific explorations of the factors affecting or predicting athletic achievement have a long history. In 1869, Francis Galton [2] described what might be the first study of sporting ability in his highly influential book *Hereditary Genius*, where he discussed the tendency for success in rowing and wrestling to run in families. Since this work, entire fields of study (e.g., sport psychology, sport science) have emerged, focusing on understanding the various individual, environmental, and technical elements underpinning the remarkable performances seen in contemporary sport.

Traditionally, these factors are broadly grouped into the nature/nurture dichotomy, with nature reflecting innate or genetic influences on individual development, while nurture collectively refers to the influence of experience, training, opportunity and so on. Although it remains a simple approach for organizing the various influences on human development, researchers have largely moved away from this dichotomy, due to its oversimplification of the relationships between these variables (see [3, 4]). Most researchers now agree that human performance in its various forms reflects an incredibly complex interaction between an individual’s genes and their environments.

However, despite the difficulties in understanding this complexity due to the sheer number of variables involved, important discoveries about athlete development have been made. One of the most robust discoveries highlights the importance of appropriate training environments for athletes to train and compete in [5]. Ideally, these environments are matched to an individual’s developmental and learning needs, although the processes underpinning how these needs evolve over developmental time is unclear. Another highly replicated discovery relates to the extent to which researchers can quantify the genetic variation in a population (i.e., for determining heritability coefficients), leading to such accomplishments as the Four Laws of Behavior Genetics [6, 7] including:

1. All human behavioral traits are heritable.
2. The effect of being raised in the same family is smaller than the effect of genes.
3. A substantial portion of the variation in complex human behavioral traits is not accounted for by the effects of genes or families.
4. A typical human behavioral trait is associated with many genetic variants, each of which accounts for a very small percentage of the behavioral variability.

Importantly, these laws appear robust and valid despite not being able to identify the specific genes responsible for this variation [8, 9]. All this to say, while researchers have made significant advancements in the understanding of athlete development, there is much we do not know.

As researchers have expanded the range and precision of variables examined in studies of athletic populations, and clarified the definitions used to capture important outcomes, a persistent issue is the considerable variance left unaccounted for in statistical models. For instance, one of the strongest predictors of attainment historically has been time spent in practice (see [5]). However, at elite levels of attainment, this variable appears to explain very little variation in performance (1% in elite sport performers; [10]). Similarly, even in groups with the largest amount of variance accounted for by practice (e.g., 26% for games like chess in the Macnamara et al. [11] meta-analysis), most of the variance remains unaccounted for. Logically, the next question is ‘What *does* account for this unexplained variation?’. It could be that researchers are simply not measuring the most important predictors (i.e., they are not looking in the right place) and that, in the case of quantity of practice, there is so little variation in training hours at the elite level that it has no predictive utility. A solution would be to expand the range and diversity of variables being considered. Alternatively, it could be that existing measures are relevant but currently do not have the precision necessary to be able to quantify differences between groups in the elements of performance that matter (e.g., they are not sensitive or specific enough in how they assess predictors and/or outcomes; [12]). These concerns seem to be those most often considered by researchers and sport stakeholders.

The notion that problems with prediction can be resolved with more, and better, data also has a long history. In one of the most influential early treatises in probability theory, Laplace [13] proposed that with sufficient knowledge and intellectual capabilities, it would be possible to devise formulae to predict any outcome. This approach, known as *Laplace’s Demon*, reflects the most extreme form of scientific determinism, based on the assumption that meaningful variables in any relationship are infinitely knowable and our capacity to predict future states is constrained only by a lack of understanding (i.e., limits of theory) and the current limits of precision (i.e., limits of measurement).

Alternatively, it might be that the process of development and, ultimately, athletic performance, is governed by variables that operate in ways that are hard (or impossible) to capture in traditional statistical approaches. It is this last element that is the focus of this paper. More specifically, we examine the role of chance and randomness (i.e., events where there is a low probability of occurrence) in athlete development, focusing on the influence of ‘luck’ on this process.

### The luck problem

While luck can be both positive (good luck) or negative (bad luck), to simplify the discussion we have focused on the positive element of this concept, as seen *when an individual gains some type of advantage over their peers through no direct (i.e.*,* conscious) engagement of their own*. Previous research integrating luck into discussions of exceptional achievement can generally be grouped into two categories. The first category of research includes conceptual models of talent development such as Gagné’s Differentiated Model of Giftedness and Talent [14] or Simonton’s Emergenic and Epigenetic Model [4] that imply (Simonton) or explicitly state (Gagné) the role of varying random elements that just happen to work out in an individual’s favour. Even in models of human development that focus on the role of practice in explaining skill and achievement (e.g., Ericsson et al.’s Deliberate Practice Framework [15]), luck may be a central factor. Access to learning resources such as competent instructors and high-quality training facilities [5], not to mention supportive parents, siblings, and peers [16, 17], undoubtedly plays a role in promoting engagement and skill development, but how does a child end up in these types of environments (e.g., as the later-born sibling in a sporty family or during the appropriate zeitgeist) if not by luck?

The second, and much larger, category of studies has explored relationships between luck and success mathematically. For instance, several research teams have used computer simulations to model the influence of luck versus talent on the likelihood of an outcome occurring over time (e.g [18–20]). This work proposes that successful individuals in a population are more likely to have an average amount of talent coupled with being the recipient of sequences of favourable events. While based on computer simulations framed around outcomes outside sport and athletic performance, these models highlight an intriguing assumption – that luck plays a key role in limiting or promoting achievement and success (e.g., by influencing athletes’ access to the types of key developmental resources mentioned above). These explorations have not been limited to simulations; for example, several studies have used historical data to consider the impact of luck in poker [21], golf [22], and basketball [23].

### Types of luck

The types and forms of luck affecting athlete development are wide and varied, especially considering the extensive timeframe of athlete development and the complex matrix of variables that interact to influence an individual’s likelihood of sporting success. In the section below we provide examples of known elements of randomness that affect athlete development relative to three broad categories: luck related to elements of the task being performed, luck related to the athlete developmental environment, and luck related to biological processes.

#### Luck related to elements of the task being performed

Previous discussions of luck in sport have generally focused on how elements of chance, low probability events, and randomness affect aspects involved in the task itself. These can include coin flips used at the start of a match to determine task-related aspects such as who kicks/receives the ball in American Football, who gets which side of the court in volleyball or who serves first in tennis. Various scholars have categorized the different ways chance events can influence sport-related outcomes [23–25]. For example, Kobiela [26] proposed five categories to capture the way chance influences sport-related tasks. The first, *aleatoriness*, reflects the purely random elements that are nonetheless built into the constituent rules of the sport (e.g., the coin flip examples noted earlier). *Chaos*, the second type of chance, involves the subtle but meaningful effects such as when a ball hits the net in tennis and lands on one side or the other, providing advantage or disadvantage in an unpredictable way. *Irregularity* involves the ‘corruption’ of elements of the game such as when officials make incorrect judgements or when weather influences aspects of game play. The fourth type of chance, *imprecision*, describes the gaps between intention and successful execution. Even the most skilled performers will experience situations where a sound strategy does not end up in a successful result due to variations or imprecision in elements of motor control in executing the strategy. Finally, *arbitrariness*, relates to the seemingly indiscriminate decisions a player might make in competition (e.g., in the absence of complete information, choosing to pass to player X, which ended up being the appropriate decision).

#### Luck related to elements of the athlete development environment

While it appears many aspects of sport have been purposefully designed to integrate elements of chance, arguably to make it more interesting and less predictable, one area where the chance element is less welcome pertains to how individuals access, and are treated within, developmental environments. Creating systems of development and competition that are equitable, fair, and balanced assumes, at least to some extent, that differences between individuals can be managed by the system or do not have significant influences on the outcomes that matter. Unfortunately, neither of these assumptions appear to be supported. A range of persistent biases have been identified in studies of athlete development ranging from socioeconomic biases (i.e., advantaging those from higher socioeconomic groups [27, 28]) and geographic biases (i.e., advantaging individuals from specific countries [29, 30] or geographic regions of a certain size [31]), to age and maturity-related biases (e.g., usually advantaging those who are maturing at a faster rate or with greater ‘relative age’ [32–34]). In these examples, those who are ‘lucky’ (i.e., on the advantaged side of the comparison) generally gain access to greater developmental resources (e.g., better competition and training environments) not provided to their unlucky peers. Moreover, in sports with early talent identification and ‘streaming’ at youth levels, being the beneficiary of early luck may provide disproportionate advantage compared to later luck, since early opportunities that increase the quality of training and competition are compounded over time.

#### Luck related to elements of biological processes

The final category of luck is the one most proximal to the athlete and involves random elements related to individual biological development. At the most fundamental level, these include the stochastic elements found at the cellular level driven by the nonlinear inter-molecular interactions involved in cell function, and the thermodynamic instability of the environments in which cells operate [35]. Similarly, proportions of different genes vary from individual to individual, resulting in the considerable variability we see in human populations. In some cases, this genetic variability can affect an athlete’s likelihood of success in sport. For these factors, the variability is purposeful as the unpredictability of these elements has an evolutionary purpose by adding variation to populations for the purpose of adaptation [36]. Other sources of variation are less purposeful, as when prenatal development is affected by the mother’s nutrition [37], alcohol intake [38], and/or exposure to viruses or pollutants [39], among other factors. These effects have long-lasting influences on overall development and risk of certain diseases (see [40]). It is almost certain they affect athlete development as well, although work in this area outside of sport genetics research is essentially non-existent.

### Getting lucky: can we manufacture luck?

Does embracing the notion of luck in athlete development and performance further confuse an already complicated area? Why should stakeholders concern themselves with integrating luck into models of athlete development or approaches to coaching and athlete selection? The reality is, random elements that provide advantages to some are ubiquitous, constantly affecting our potential for success and development, so any model that omits these elements will be fundamentally incomplete.

However, the implications of integrating a role for luck in explaining athlete success are more wide-ranging. For instance, acknowledging the influence of luck shifts the conversation from the traditional debate about nature versus nurture, where elements can be understood, explained, and predicted, to one where a certain degree of randomness is foundational to how we need to view the complex processes of athlete development, competitive success, injury avoidance, and so on.

Acknowledging a role for luck in athlete achievement does not mean performance and success become an unpredictable, convoluted mess of interactions impossible to decipher. It simply recognizes that the many influences on athlete development and performance vary in the extent to which they are predictable. For example, many performance-related variables (e.g., height, aerobic fitness, technical skill) would be largely stable between competitions in normal circumstances (e.g., barring injury or other disruption) while others (e.g., life stress, environmental conditions, quality of one’s opponents) are more variable, affecting performance in less predictable ways. For longer term outcomes, the time course involved decreases the capacity to predict outcomes in meaningful ways over time as nearly all variables will change over the course of development.

One way to consider the impact of luck would be to separate the elements that are uncontrollable and random from those that are the result of policy or system-related factors that are open to manipulation. Considering luck in this way would distinguish pure randomness from system-design elements that differentially affect an individual’s likelihood of success. While elements of ‘uncontrollable variability’ are still relevant to future success, they are outside the control of coaches, policy makers or other stakeholders, while other elements (e.g., ‘controllable variability’) could be managed through policy change, changing system structures, and education. A starting point would be distinguishing these types of luck from each other. For instance, Aicinena [24] distinguishes between moral luck (i.e., elements of an individual’s life over which they have no control that affect their development) and simple luck (i.e., uncontrollable events that impact the outcome of a contest or success over the course of a season). However, for coaches, policy makers, and administrators, the most relevant distinction could be between chance events and serendipitous ones. *Chance* could be used to describe something that happens unpredictably without discernible human intention or observable cause and is much harder to predict, while other forms of luck such as being in the right place at the right time could be grouped together to reflect a kind of s*erendipity* (i.e., luck in the form of finding valuable or pleasant things that are not explicitly looked for). For many sport stakeholders, serendipity is the variable of greater interest since it is the category of luck with greatest potential for change/improvement. These improvements can come from two sources: increasing opportunities for serendipity to occur and increasing measurement precision to reduce the need for serendipity in the first place.

### Increasing opportunities for serendipity

Increasing opportunities for serendipity in athlete development starts with an understanding of how the current system promotes inequality of opportunity through seemingly random elements. Rigid pathway models and coaching philosophies may increase the potential for luck to play a significant role in an athlete’s development. For instance, approaches to athlete development that emphasize early streaming and selection, as well as siloed approaches to interactions between sports (e.g., when coaches in one sport do not want their athletes to play other sports), put a greater emphasis on the role of luck that athletes who begin the pathway are in the right place at the right time. From this perspective, one potential solution to the luck problem would be to design systems that do not limit opportunities for athletes in their development and devise ways to create even more opportunities than currently available. Early approaches that emphasize participation in multiple sports often rationalize this as being beneficial because this form of engagement is more likely to meet the needs of fundamental skill development and playful engagement, but a more varied exposure to multiple sports also helps mitigate the role of luck by exposing athletes to multiple domains where they can explore their future potential.

### Improving precision

What would seem to be the counter position is a focus on improving approaches to athlete assessment and building stronger models to capture the complex process of athlete development. One of the reasons luck plays such a meaningful role in the current context of athlete development is because current approaches to athlete identification and development have significant limitations. As noted above, fixed systems of development where athlete engagement is through a single sport increase the influence of luck by making the process of finding the proper fit between athlete and sport dependent upon a small number of opportunities. While increasing the opportunities is one way to increase the chances of getting ‘lucky’, another option would be to remove the need for this luck in the first place.

**Fig. 1:**
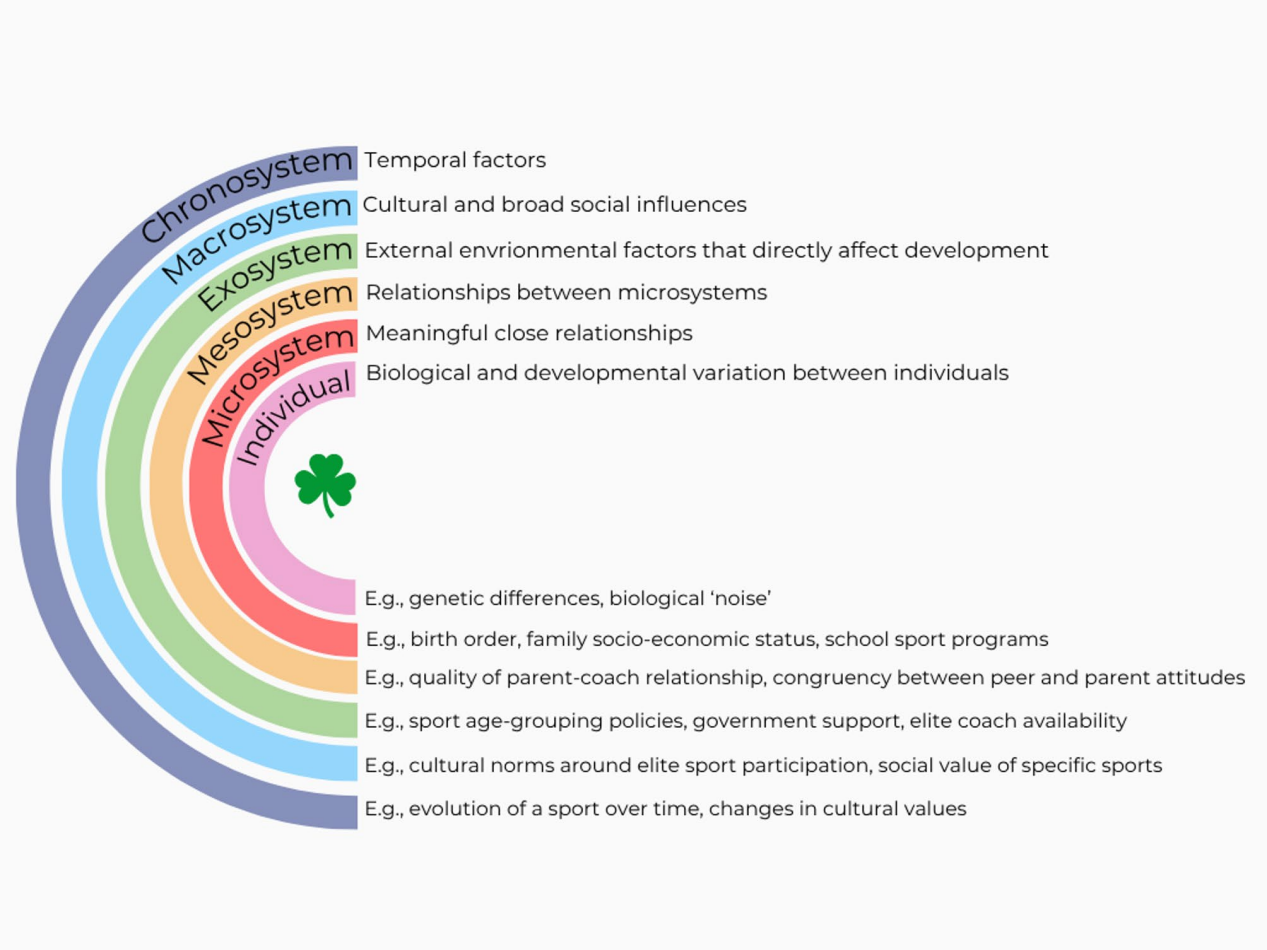
Examples of luck across the systems in Bronfenbrenner’s Ecological Systems Theory [41]

Several areas of athlete development could be improved through superior approaches to measurement. For instance, a better understanding of individual (e.g., intrinsic athlete motives) and environmental factors (e.g., availability and accessibility of different activities in an athlete’s environment) could facilitate probabilistic models of success, which could be used by sport systems to improve the provision of important resources (e.g., facilities, coaches) that end up affecting the interest of the individuals in the environments where these resources are provided. Similarly, better understanding of how development- and performance-related variables change across time would allow for the construction of more accurate models of long-term developmental success. At the same time, it would be prudent to consider how an increase in measurement precision would affect the long-term sustainability of high-performance athlete development models. For instance, if we were able to predict an athlete’s likelihood of success at a young age, how would this affect their motivation for long-term engagement (e.g., if it were possible to tell an athlete they have no chance of Olympic success, would this be a positive thing for their long-term development and wellness; would it be beneficial for the system as a whole?)? From this perspective, the need for luck arises from the value of uncertainty in sport and in athlete development systems (i.e., we do not want sport to be too predictable). We have tried to capture these sources of uncertainty in Fig. 1. Bronfenbrenner’s Ecological Systems Theory [41] was chosen as the foundational model, because of its emphasis on the complex, multi-layered influences on human growth and development. Elements of luck can be meaningfully and easily embedded at every level. For example, it could be considered lucky if an athlete was born with a preferred combination of genes that lead to a phenotype a coach was looking for in a given sport (i.e., individual level), and when an athlete is born into a family that can afford sport participation when the athlete is of the best age for that participation to begin (i.e., microsystem). Similarly, having strong coach-parent relationships, being born in the first quartile of a sport’s selection year (i.e., relative to a cutoff-date), and being attracted to ice hockey in a country with many ice rinks reflect luck’s influences on elements of the mesocycle, exocycle, and macrocycle levels. Finally, having all these elements occur at the right moment in time when these specific opportunities are offered and characteristics are valued reflects a form of temporal luck (i.e., the chronosystem).

Further, increasing precision in athlete development contexts is not simply about ‘doubling down’ on measurement through an increase in the number of variables or indicators used to try to find where an athlete fits. Precision also stems from a recognition of where the limits of our ability to predict end. Given our incomplete understanding of the process of athlete development and the poor-quality evidence across much of this field (e.g., cross-sectional studies focusing on a limited range of samples [42, 43]), coupled with meta-analytic reviews on the disconnect between early achievement and later success [44], the position to support early athlete streaming or actively limit an athlete’s sport exposure during youth is surprisingly weak considering how entrenched these approaches have become in many countries.

### Challenges to integrating luck into models of athlete development

Even if there was widespread agreement that many aspects of athlete development have an element of randomness and unpredictability, integrating this concept into athlete development would not be easy. The cultures of many sports reinforce philosophies of athlete development that under-appreciate the role of luck in explanations of athlete development. For instance, frameworks such as deliberate practice place individuals at the top of a hard work meritocracy where skill acquisition results from time spent in focused, intensive, training. These approaches might undermine the creation or maintenance of environments and structures that facilitate that luck in the first place because they place the emphasis (or too much of the emphasis) on factors that do not deserve it [45]. Rather than having individuals work from a position of “I got mine through hard work; you work hard for yours!”, it is more equitable to have individuals acknowledge the random influences that they were the beneficiary of, so that they can promote and facilitate those same factors in others. However, doing so would require overcoming several challenges.

First, the dominant approach to athlete development currently reflects the assumption that successful outcomes are the result of a predictable pattern of interactions between knowable variables. From this perspective, coaches, parents, athletes and other stakeholders just need to ensure they ‘get things right’ at each stage of development. Recognizing the influence of luck would require acknowledging a degree of unidentified (and likely unidentifiable) variation in athlete development processes. Second, acknowledging this influence may be possible at the theoretical and conceptual levels, but also needs to be integrated into how coaches and athletes (as well as other interest-holders) see their performance and development. It may be difficult for learners to separate themselves from the obvious hard work and effort they invested into their development to give a meaningful role to luck. Instituting meaningful change in coaching practice or athlete development policy will require overcoming any forms of self-serving attributional bias (e.g., narrative bias, hindsight bias). Finally, the nature of the influence of luck is hard to ‘prove’ empirically. While it is reflected in forms of statistical error and unaccounted for variance, it remains nebulous. Given the ways chance and randomness affect different aspects of human development, it will never be possible to analyse the process of athlete development and see ‘where’ the luck happened. This makes the idea of luck hard to integrate into discussions with scientists and practitioners whose roles are to explain and predict.

## Conclusion

Despite these challenges, appreciating a greater role for luck may be important for improving discussions of athlete development in many sports. Notably, the types and forms of luck likely morph and evolve across development (see Mauboussin’s notion of the ‘Paradox of Skill’ [46]). For instance, later in the athlete development system, when athletes have become more homogeneous relative to key performance variables (e.g., height in basketball players), luck may be more meaningful since important sources of variance in performance are harder to determine. From these perspectives, acknowledging the role of luck may lead to sporting environments that are more equitable (by creating greater opportunities for more individuals to get lucky) *and* realistic (i.e., by acknowledging that predictions of sport- and athlete-related outcomes will never be perfect). This does not imply we should ignore elements of athlete development where greater precision is necessary. Instead, it promotes the need to determine the limits of measurement precision. This distinction is important in the current data-intensive culture of high-performance sport.

## Data Availability

Not applicable.
